# Experiments on MEMS Integration in 0.25 μm CMOS Process

**DOI:** 10.3390/s18072111

**Published:** 2018-06-30

**Authors:** Piotr Michalik, Daniel Fernández, Matthias Wietstruck, Mehmet Kaynak, Jordi Madrenas

**Affiliations:** 1 Nanusens, Av. del Parc Tecnològic 3, CENT – Parc Tecnològic del Vallès, 08290 Cerdanyola del Vallès, Spain; piotr.michalik@nanusens.com (P.M.); daniel.fernandez@nanusens.com (D.F.); 2 IHP, Im Technologiepark 25, 15236 Frankfurt (Oder), Germany; wietstruck@ihp-microelectronics.com (M.W.); kaynak@ihp-microelectronics.com (M.K.); 3 Electronic Engineering Department, Universitat Politècnica de Catalunya, Jordi Girona 1-3, 08034 Barcelona, Spain

**Keywords:** CMOS-MEMS, CMOS, MEMS, BEOL (Back End of Line), accelerometer

## Abstract

In this paper, we share our practical experience gained during the development of CMOS-MEMS (Complementary Metal-Oxide Semiconductor Micro Electro Mechanical Systems) devices in IHP SG25 technology. The experimental prototyping process is illustrated with examples of three CMOS-MEMS chips and starts from rough process exploration and characterization, followed by the definition of the useful MEMS design space to finally reach CMOS-MEMS devices with inertial mass up to 4.3 μg and resonance frequency down to 4.35 kHz. Furthermore, the presented design techniques help to avoid several structural and reliability issues such as layer delamination, device stiction, passivation fracture or device cracking due to stress.

## 1. Introduction

In the last decades, MicroElectroMechanical Systems (MEMS) have demonstrated a steady trend of miniaturization as well as cost and power consumption reduction, while maintaining or improving performance. This triggered their wide use in high-volume consumer electronics devices such as smart phones, tablets, video game consoles or wearables mostly as sensors such as accelerometers, gyroscopes, magnetometers or pressure sensors to name the most common ones. Nevertheless, practically all the commercial implementations require a custom MEMS fabrication process, which is inconvenient from cost, fabrication and packaging points of view. For example, the commercial MEMS accelerometers and gyroscopes are either multi-chip modules or at least a separate MEMS process is applied to the same wafer to deploy the mechanical device next to the electronics or on top of a CMOS chip.

CMOS micromachining, where standard CMOS back-end of line (BEOL) layers are used as MEMS structural and sacrificial materials, has been a promising approach for many years. Previously, several devices based on anisotropic etching of full interconnection stack followed by isotropic [1] or Deep-Reactive Ion-Etching (DRIE) [2] substrate etching have been reported. More recently with the advent of Analog/ Radio-Frequency (RF)-oriented microelectronic technologies, significant effort has been made to develop RF-MEMS devices such as switches [3] or resonators [4] by using simple isotropic etching of inter-metal dielectric (IMD) of the CMOS BEOL [5,6]. This method offers possibly a very cost-effective solution as only simple post-processing is needed.

In the case an RF-oriented process is applied, thanks to thick metal and via layers used normally for inductors or power routing, more bulky devices such as inertial sensors can be obtained by releasing the top thick metal layers, what we demonstrated in [7,8]. Furthermore, if sufficient number of metal layers is available, the same area can be shared between MEMS and electronics, by placing the electronics below the MEMS.

In this paper, we share our practical experience gained during the development of CMOS-MEMS accelerometers in IHP SG25 technology. While different devices obtained by isotropic oxide etching of the CMOS BEOL stack can be found in the literature, the available publications are usually focused on the final results and there is relatively little papers that reveal the path to functional device implementation, especially regarding possible failures.

Using as examples three CMOS-MEMS chips that we taped out, we demonstrate a gradual improvement of the achieved results by eliminating the main manufacturing issues and taking advantage of the fabrication process properly. In Section 2, the CMOS process (with the emphasis on its back-end part) and the MEMS post-processing steps are briefly described. Then, Section 3 introduces the research methodology illustrated with a short overview of three developed CMOS-MEMS chips. In Section 4, a qualitative properties of the process such as etch rates, stress behaviour, layer stacking and design techniques addressing the main manufacturing problems are presented concluding with a definition of the useful MEMS design space. Section 5 presents a selection of simulations and measurements of functional MEMS accelerometers with a comparison of their performance. Finally, in Section 6, the conclusion is stated.

## 2. Technology

### 2.1. IHP SG25 Process

**Figure 1:**
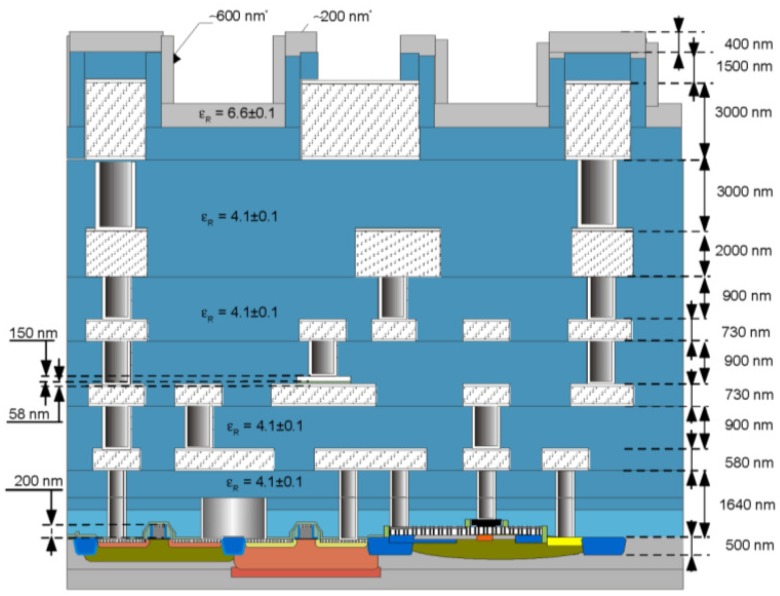
IHP SG25 process cross-section (taken from [9] with permission).IHP SG25 process cross-section.

A simplified cross-section of IHP SG25 process is presented in Figure 1. Principal applications of this SiGe BiCMOS technology are RF and microwave circuits. Nevertheless, only the BEOL and the standard 0.25 μm CMOS module were used in our research.

The BEOL part of the process consists of five metalization layers made of AlCu (typically, around 0.5% Cu used to improve electromigration robustness [10]) stuck between two Ti/TiN caps that serve as tungsten diffusion barrier and antireflection coating (ARC) [10] and despite their small thickness (typically 50–100 nm) have important impact on the mechanical properties of each layers, due to their stress contribution and very high Young’s modulus [11]. The top two layers—TM1 and TM2 are 2 and 3 μm thick, respectively, and are separated with 3 μm thick tungsten via (TVia2). As shown below, such a configuration provides excellent possibilities to develop suspended devices of relatively big mass and high thickness—a key factor to achieve a good sensitivity in the case of accelerometers and other inertial sensors. The thickness of the other metal layers is below 1 μm. Besides, there is a Metal-Insulator-Metal (MIM) capacitor available on M2.

### 2.2. MEMS Post-Processing

**Figure 2:**
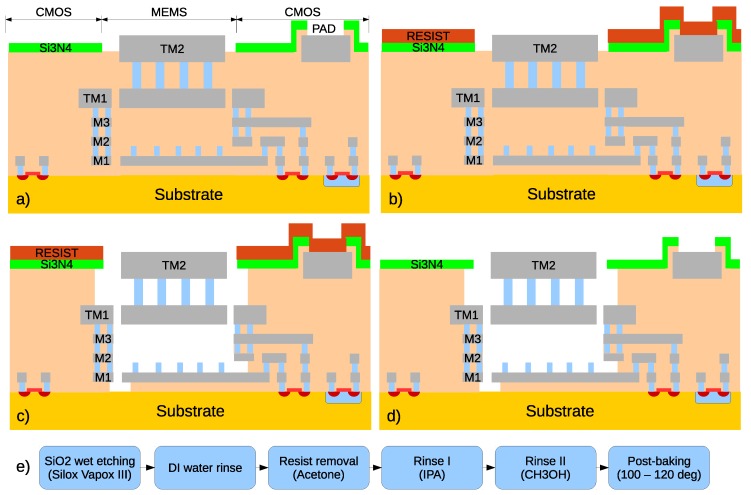
Simplified cross-sections illustrating the post-processing steps: after standard CMOS processing finished with the passivation opening (**a**); after the photo-resist deposition to protect the pads and passivation (**b**); after the SiO2 wet etching (**c**) ;and after the resist removal, rinsing and post-baking (**d**). The diagram (**e**) enumerates the steps used in the in-house release process.

In Figure 2, a simplified cross-section of a CMOS-MEMS chip during the subsequent post-processing steps is depicted. In this particular case, the TM1-TVia2-TM2 structure is suspended over M1 fixed plate covered with via dimples. In reality, we tried different layer stacks and the etching depth depended on the attack duration.

The post-processing starts with a CMOS wafer after the final standard fabrication step of passivation opening (Figure 2a) which is covered with photo-resist to protect the pads and the passivation (Figure 2b). After that, a non-thinned (for easier sample handling) wafer is diced and individual the samples are shipped, so that the final in-house processing is performed with the following steps (Figure 2c–e):

1. Wet oxide etching using Silox-Vapox III pad etchant;
2. Deionized water rinse;
3. Resist removal in acetone;
4. Isopropanol and methanol rinse (as liquids with a very low surface tension, they were used as a low-cost replacement for critical-point drying); and
5. 20 min at 100–120 C bake.

The samples to be released were handled manually and individually transported between the subsequent etching and cleaning agents, so that the release time of each chip could be controlled independently. In further trials when the etching characteristics was known better and the needed release time was defined more precisely, multiple samples were glued to a carrier wafer with a resist and handled simultaneously.

Silox-Vapox III from Transene Inc. (Danvers, MA, USA) is characterized by improved aluminum selectivity and is a combination of the following: ammonium fluoride, glacial acetic acid, aluminum corrosion inhibitor, surfactant and deionized water. The following reactions occur during the etching process:

$$\begin{array}{c} {\mathrm{CH}}_{3}\mathrm{COOH}+{\mathrm{NH}}_{4}F\;\;\;⇄\;\;\;\left({{\mathrm{NH}}_{4}}^{+}\right){\mathrm{CH}}_{3}COOH^{-}+HF+H_{2}O\\ SiO_{2}+4HF+2{\mathrm{NH}}_{4}F\;\;\;⇄\;\;\;2{{\mathrm{NH}}_{4}}^{+}+{{\mathrm{SiF}}_{6}}^{2-}+2H_{2}O \end{array}$$

Numerous devices obtained with this etchant have been reported previously [5,6].

## 3. Methodology and an Overview of the Prototype Chips

Traditional MEMS technologies, where process properties are well controlled, optimized and the design rules are known, allow basing essential part of the prototyping process on computer-aided simulations. In the case of MEMS implementation within CMOS BEOL, usually few reliable (from micromachining point of view) process data and design experience are available. The process recipes are industrial secrets and their modifications are very expensive as they require process requalification and since they may compromise CMOS yield, in the case of multi-project wafer (MPW) runs they are simply unacceptable. These two issues conditioned our prototyping strategy to be the following:

1. Trial-error was chosen as the most suitable prototyping method.
2. The devices had to be adapted to the process, not the other way around.

Following this approach, we taped out three chips: *Accused* with the first test structures that served for “reconnaissance by fire”; *Bailed* that allowed more accurate design space exploration and was a proof of concept of MEMS monolithic integration with electronics; and *Released* with refined design of low-noise CMOS-MEMS accelerometers that reached state of the art performance.

### 3.1. *Accused*—Reconnaissance by Fire

**Figure 3:**
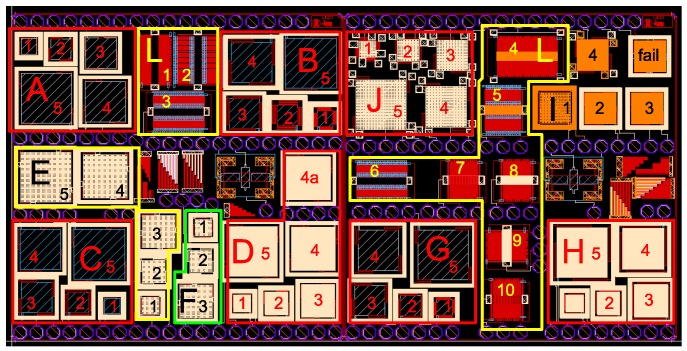
*Accused*—the first CMOS-MEMS test-chip in IHP SG25 BEOL-only process. The dimensions are 2 mm × 4 mm.

In Figure 3, the layout of our first CMOS-MEMS test-chip implemented in IHP SG25 BEOL-only technology is depicted. Besides numerous single- and multi-metal cantilever test structures, it comprises several groups of devices (A–J and L), where each group contains a series of devices of similar construction and layer configuration, but of various sizes. Devices from Groups A–I are parallel two-plate MEMS capacitors (prototyping platform for single-ended *z*-axis accelerometer), and Group J includes three-plate MEMS capacitors (prototyping platform for differential *z*-axis accelerometer). Both are inspired by vertical accelerometers presented in [12]. Group L is a prototyping platform for lateral *x*/*y* axis accelerometer and comprises various lateral comb MEMS devices with classic differential electrode arrangement similar to ADXL50 [13].

**Figure 4:**
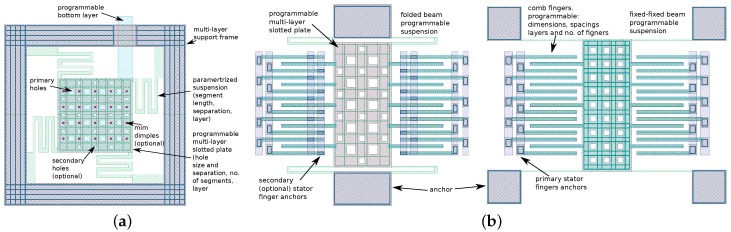
Parameterized cells examples: *z*-axis two-plate device (**a**); and two cases of lateral *x*/*y*-axis devices with folded beam and fixed-fixed suspension (**b**).

For the purpose of rapid prototyping, those devices were implemented as parameterized cells (pcells) with programmed dimensions, suspension and layer compositions. More detailed layout examples of two-plate and lateral devices can be seen in Figure 4. The layer compositions of the device groups deployed on the chip can be seen in Table 1 and Table 2. As it can be seen, M3 was used most widely as a structural layer on the chip, which was an effect of the inspiration with IHP RF-MEMS switch [11] that was built with that layer.

**Figure 5:**
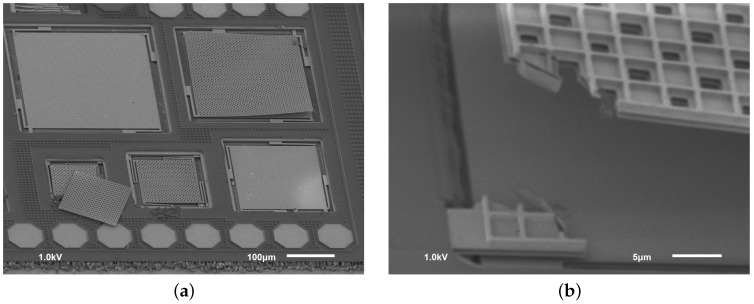
Typical failures on *Accused* chip: delamination of TM1 layer, contamination with metal fillers from the cut area and corner cracks (**a**). Zoomed corner (**b**) reveals that multi-layer plate is released before the suspension and the corner, weakened by the perforations, cracks due to stress.

Unfortunately, it was not found to be a correct decision. First, the stress programmed in M3 layer to increase the reliability the RF switch seriously limits the maximum dimensions of M3 structures. Furthermore, the adhesion between the TVia1 (via on top of M3) and TM1 layer was found to be insufficient to guarantee multi-layer structure integrity after the release process. For these reasons, most of the tried devices simply failed, while only a few (F1 and F2) could be correctly measured electrically [14]. Another issue related to MEMS post-processing of discrete dies coming from a MPW wafer was chip contamination by the metal fillers released from the die cutting area (see Figure 5).

Despite all those problems, the chip provided a great amount of information on how to use the process efficiently and was a milestone for further developments, as its principal objective was “reconnaissance by fire”—getting as much information as possible on the MEMS release process behaviour, checking feasibility of applying various metal layer combinations and determining the useful range of their dimensions.

### 3.2. *Bailed*—Focus and Proof of Concept

**Figure 6:**
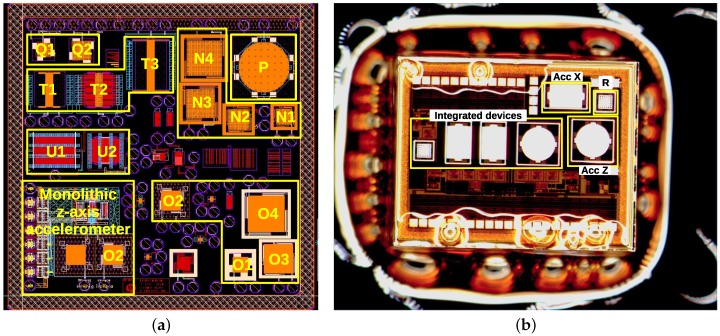
Layout of *Bailed* chip fabricated in SG25H3 full process (**a**). The dimensions are 2.2 mm × 2.2 mm including the 200 μm wide metal guardring. Microphotography of 2 mm × 2.5 mm *Released* chip (**b**). The manually applied photoresist is used to stick the sample to a carrier wafer and is deliberately covering the pads to correct the etching mask error.

*Bailed* was submitted to explore other layer combinations with emphasis on TM2 layer and TM1-TVia2-TM2 stack. An overview of the layout can be seen in Figure 6a. A big multi-metal guard-ring surrounds the chip to be used as die cutting area so that contamination with fillers released from the cut area is avoided. At the same time, the global metal density requirements for the chip interior are also relaxed.

Mainly different configurations of the *z*-axis pcells used in *Accused* chip were used (see Table 1), however they were slightly refined: rounded instead of straight angle suspension meanders and via instead of MIM dimples were applied. In addition, some of the release holes were skipped in order to enforce the weak points at the corners. Besides the square devices, a circular plate of 360 μm diameter (P) suspended on eight meander springs was added. In the case of the in-plane devices, the previous pcells were also reused with minor modifications and different layer configuration (see Table 2).

One of O2 *z*-axis devices was integrated with a simple conditioning circuitry becoming the proof of concept of CMOS-MEMS monolithic integration [7]. While the performance of this device was quite weak due to very conservative design (20 kHz resonance), the other devices on the chip revealed much greater potential of the technology reaching 4.4 kHz resonance in the case of O4 device and demonstrating a first successfully released lateral device (T1) with resonance frequency of 33 kHz (see Section 5 for more details).

### 3.3. *Released*—Monolithically Integrated Accelerometer and Resonance Pressure Sensor

Based on the experience gained from the previous chips, the final chip, *Released*, was taped out. It comprises *z*-axis acceleration sensor based on circular device P from *Bailed* chip, as well as the final in-plane (*X* or *Y* axis) acceleration sensor with 7.4 kHz lateral resonance [8]. Besides, a 100 kHz resonator for resonance pressure sensing [15,16] is also used to check the process stability. All the MEMS utilize the same TM1-TVia2-TM2 stack for the mechanical structure with one modification—the top Ti/TiN ARC from TM2 is removed. The chip integrates state-of-the-art capacitive sensing front-ends and each MEMS device appears in an integrated and a test version.

## 4. Process Characterization

### 4.1. Silicon Oxide Etching Rate

By using variable attack duration, samples with different number of released metal layers were obtained—from only TM2 release to removal of the total oxide down to the Si substrate. In Table 3, the approximate release depth in a function of time is expressed with three possible states of a layer—*covered* with oxide, *exposed* (but not yet released) and *released*, which in the Si case refers to cleaning out all the oxide from the substrate within the MEMS area.

**Figure 7:**
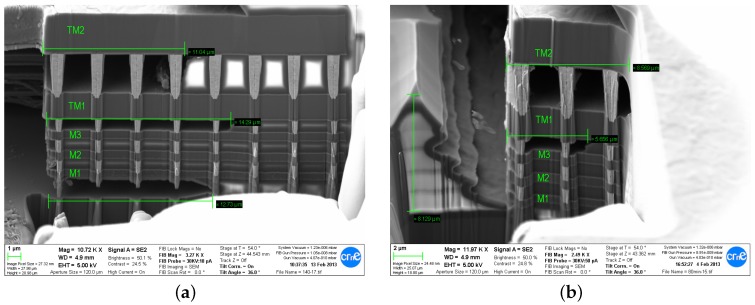
FIB cut of multi-metal guardring after: 140 min etching (**a**); and 80 min etching (**b**). Note that the etching advanced much faster close to the metal wall. In addition, TVia1 and TVia2 via have not stopped the lateral oxide etching.

Silox-vapox III is designed for deposited silicon oxide etching and the producer claims that a typical etch rate is 4000 Å/min. Nevertheless it is well known that the actual etch rate of silicon oxide strongly depends on the deposition process and the appearance of different dopants and impurities [17]. According to Dai [5], the etch rate of inter-metal dielectric in TSMC 0.35 μm technology was 960 Å/min. Our experiments showed also a rate around 1000 Å/min as the ∼13 μm thick oxide stack is etched down to substrate between 120 min (silicon is visibly exposed, but not entirely uncovered) and 140 min (no oxide left in a MEMS cavity as in Figure 7a).

While the etch rate was relatively repetitive over numerous experiments on chips from different runs and wafers performed over several years, in some cases, it was found to be increasing for long reaction time. In one of the experiments, 80 min etching was planned to correctly release the MEMS structures, however, the samples, instead of being immersed in a fresh etchant, were added to a container where several other chips had already been etched for around 40 min. As a result, all the added samples were severely overetched and the MEMS devices that otherwise would be functional were damaged.

#### 4.1.1. Etching Isotropy

The actual etch rate is not perfectly isotropic and depends strongly on the geometry of the etched area. The etching reaction appears to be catalyzed in the proximity of the metals as it was found to be significantly faster close to the metal obstacles in either vertical or lateral etching directions (see Figure 7b). Similar phenomenon had been reported in the case of gaseous HF etching of CMOS-MEMS structures in [18].

Such inhomogeneity of the etching results in faster releasing of thick layer stacks than standalone metal layers of the same depth, which in some cases may have a devastating effect on a MEMS device, as it can be seen in Figure 5, where the multi-metal MEMS would be already released, if the M3 suspension was not still covered with oxide. In that case, the plate corner fixed by the unreleased suspension has not resisted the excessive stress and cracked. In the following chips, the release holes close to the corners were skipped in order to reinforce the device strength.

Similar phenomenon was observed when the same metal stack was released if there was a metal plate below the released stack and unreleased if there was no metal below it, even though the released stack geometry and the holes pattern were the same.

### 4.2. Etching Selectivity against Other CMOS Materials

An ideal etchant would selectively remove the sacrificial oxide, while leaving the structural materials untouched. While this is practically true for gas HF etching and its selectivity to aluminum [6], in case of wet etching, a small etch-rate against aluminum as well other materials apparent in a CMOS process exists.

**Figure 8:**
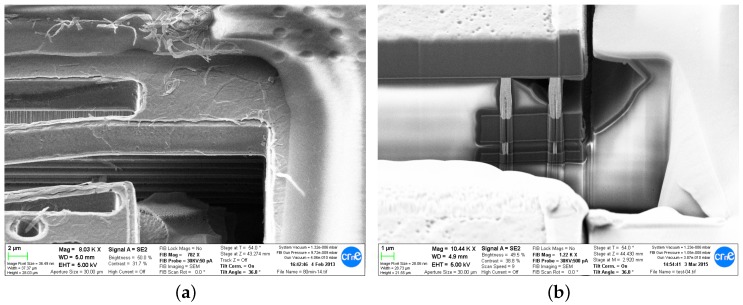
Top Ti/TiN ARC damage on TM2 layer on *Accused* chip after 80 min release (**a**); and FIB cut of an unprotected pad after 85 min release of *Released* (**b**)—the etching around the pad does not pose any danger to the electrical connections.

In comparison to other etchant compositions, Silox-Vapox III has relatively good selectivity against aluminum and makes little impact on the surface roughness. Nevertheless, it is known to have a slow etch rate of Al in range of around 10 Å/min [5], which we observed as well. On the other hand, no etching of tungsten vias has been noticed and the Ti/TiN layers were also well preserved except the top layer on TM2, which got peeled and cracked even at relatively short etching times (see Figure 8a). Due to the different etch rates, AlCu is undercut between the Ti/TiN layers that preserve their layout dimensions. This puts a limit on minimum useful structure width for a given metal layer and etching time, as in case of excessive undercut, the Ti/TiN layers tend to crack and in extreme cases the AlCu is completely removed. For the etching times around 85 min that were sufficient for *Released* chip, typical AlCu undercut was around 200 nm for TM1 layer. This permitted using suspension beams of 2 μm width. For longer etching times—in the range of 100–120 min—1 μm wide M3 suspension beams were already damaged.

A simple and very effective measure to deal with Ti/TiN damage on top of TM2 was used in the final chip, and consisted in removing Ti/TiN ARC by using the *passiv* mask around the MEMS structures, which normally is used to remove the passivation and ARC from pad area. Some vertical etching of the AlCu would typically be its only side effect. In that chip however, the *passiv* mask was also erroneously assigned to be the MEMS etching mask that defines the photoresist that protects the passivation and the pads during the release. This resulted in unwanted exposure of the pads, which normally would be protected.

#### 4.2.1. Pad Etching

While initially adding manually some photoresist on top of the pads was tried to circumvent the unwanted exposure (see Figure 6b), it was later found out with FIB examination that, for the etching times needed to correctly release the structures made of two thick metals, the oxide undercut around the pad and the pad surface damage is not severe (see Figure 8b). This shows that a common mask could be used for pads and for MEMS opening.

#### 4.2.2. Si3N4 Passivation Etching

In the case of Si3N4 passivation, it is known that its etch rate strongly depends on the layer composition and so-called “silicon-rich” silicon nitride is very resistant to pad and buffer-oxide etchants, while its “standard” version is not [17]. In the case of IHP SG25 BEOL process, “standard” nitride passivation is used, therefore a photoresist is used to protect it together with the pads. In one experiment, it was found that Si3N4 passivation of this process is indeed very sensitive to the etching, as it literally disappeared from a sample that was etched after deliberately removing the photoresist.

In addition, as the photoresist does not cover well the TM2 steps and the passivation itself can be thinner than on the TM2 sidewalls (see the profile of the chip surface in Figure 1), the etchant relatively freely penetrates the chip through the passivation in the proximity of TM2 devices as for example in case of the lateral devices in Figure 16a–c. The damage is especially severe and visible when TM2 is crossing the border of MEMS area. In the final chip, it was decided to use TM2 only inside the MEMS area and on the pads.

### 4.3. Multi-Metal Stacking

A key element to develop a functional inertial sensor using a standard CMOS process is the ability to release reliable multi-conductor stacks. Those solve or relax several issues:

- Thicker structures in general have smaller radius of curvature due to residual stress, thus a bigger device for a given vertical air gap can be obtained, which allows increasing mass and mechanical sensitivity as well as capacitance and overall capacitance sensitivity.
- A higher lateral capacitance can be obtained.
- Due to its high density, tungsten as a part of the stack can contribute significantly to the accelerometer proof mass.
- Elements of different thicknesses can be employed within one MEMS device, which provides additional design options.

In the first test-chip, we have tried various metal layer combinations and three different types of via patterns:

1. standard-size vias, typically arranged in arrays with the spacing close to the minimum allowed by DRC rules;
2. via bars (standard-size vias stretched in one direction); and
3. via mesh (combination of crossed vertical and horizontal via bars).

Other via shapes, such as vias stretched in both directions were not allowed in a multi-project wafer run.

For any given metal-via-metal combination and via pattern, we qualitatively compared whether metal-via-metal stack integrity an the oxide that is encircled by via, are preserved. The results are summarized in Table 4, showing that via arrays are the least robust, while the via meshes have the best performance. Nevertheless, none of those patterns could reliably stack TM1 over M3 and in all the cases delamination occurred between TVia1 and TM1. Consequently, in a given released microstructure, the layers from only one layer subset—M1-Via1-M2-Via2-M3(standard layers) or TM1-TVia2-TM2 (thick layers)—could be used.

Further investigation on the TVia1-TM1 connection did not result in discovering the reason for its failure. A possible solution may consist in increasing locally the size of the via [19]. It is also believed that gas HF etching would not penetrate through via bars.

### 4.4. Residual Stress

Residual stress is especially difficult to deal with in the case of CMOS-MEMS post-processes, as, due to limited thermal budget, a typical measure of annealing cannot be applied. Furthermore, in the case of BEOL micromachining, the compound structure of the used materials and their thermal expansion coefficient mismatch add yet another difficulty. As an example, aluminum has about five times bigger thermal expansion coefficient than tungsten.

Residual stress and stress gradients manifest mainly by curling of released structures, which may directly cause performance deterioration or even failure (for example, when a suspended plate touches a fixed plate). Other failure mechanisms related to residual stress are fracture, buckling or delamination.

In an isotropic thin film, residual stress can be approximated as an equibiaxial stress field [20]:

(1) $$\sigma_{total}\approx \sigma_{0}+\sigma_{1}\frac{z}{h/2}$$

where $\sigma_{0}$ is the average axial stress, $\sigma_{1}$ is stress gradient over film thickness *h* and zϵ(−h/2,h/2) has the origin at the film’s mid plane. When a MEMS structure made of such a thin film is released, $\sigma_{0}$ is responsible for axial expansion or contraction while $\sigma_{1}$ for its curvature. Furthermore, $\sigma_{0}$ may lead to structure out-of-plane rotation on the boundary between a released part of the film and its part fixed to the substrate (for example cantilever anchor) [20].

In an “ideal world”, $\sigma_{0}$ and $\sigma_{1}$ values of each layer and sublayer (i.e., AlCu, Ti/TiN, W, etc.) would be available for a MEMS engineer as a design entry for FEM simulations that would allow performing an accurate feasibility study. Such a study could consist in determining of a radius of curvature of selected layer stacks to check if a MEMS device would not be excessively curled, which would either compromise the performance or lead directly to a failure due to short-circuit. As such, detailed process data are not available and it is not trivial to extract them. In this subsection, we analyze only qualitatively stress behaviour and contribution to compare it with other reported data. Then, we proceed to the analysis of the radius of curvature for selected metal stack in order to define a design space for the studied devices.

#### 4.4.1. Qualitative Stress Analysis

A summary of the observed layer characteristics, based on the data collected from *Accused* and *Bailed* chips, is presented in Table 5. The data on the metal layers were concluded from direct observation of bending direction of cantilever and plates composed from different metal layers. As all the examined structures are in fact compounds of layers made of different materials, the structure deformation comes mainly from the stress mismatch between different layers.

**Figure 9:**
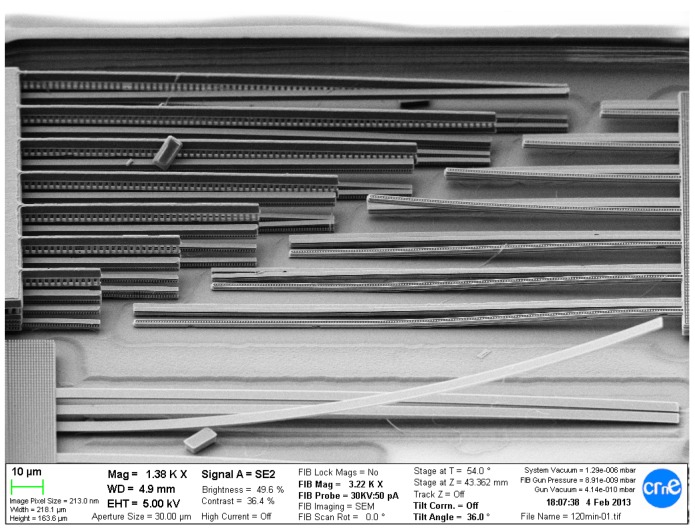
Curled-up M1 beams (left-center) showing the tensile residual stress of this layer. M2 and M3 are curled down due to compressive stress.

Except M1 (see Figure 9), all the metal membranes had convex shapes or the cantilevers were curled down, which shows a domination of negative stress gradients along the structure thickness and most probably an overall domination of compressive stress. According to the literature, thin Al and AlCu films usually exhibit slightly tensile stress at room temperature [21]. Most likely, Ti/TiN layers contribution of compressive stress and its negative gradient across the film thickness dominates tensile properties of AlCu, as it was demonstrated for M3 layer of this technology in [11].

**Figure 10:**
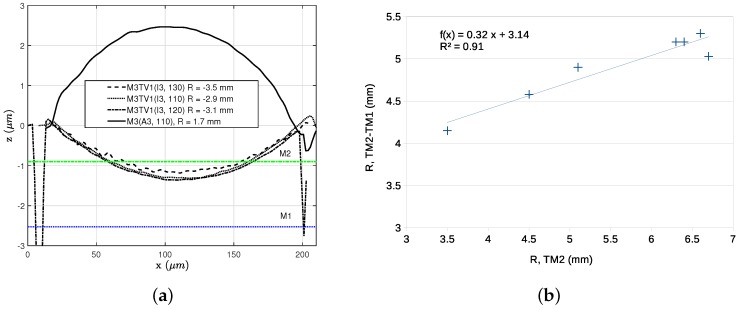
Profile and radius of curvature comparison for three different M3 —TVia1 membranes and one M3 membrane (**a**). Radius of curvature correlation for TM1-TM2(P) and standalone TM2(N4) plates obtained from seven chips (**b**).

As via beams without metal underneath were not allowed, the data on the via layers were obtained by observing the curling of metal-via compound cantilevers and plates (i.e., beams with via bars and plates with via meshes on top). In all cases, we observed concave shapes for the metal-via membranes and curled-up metal-via beams, although their metal-only equivalents had convex shapes or were curled down (example of such behaviour can be seen in Figure 10a). This shows clearly a strong tensile residual stress of all the via layers. Due to lack of a proper structure to test Via1 layer, this layer was not checked, however similar behaviour is expected. The tensile stress of tungsten films is in accordance with literature [21].

Finally, it is worth noting that, in this process, the stress behaviour is different from that reported in [22] for two other CMOS processes. In that work, both single- and multi-metal cantilevers are predominantly curled up showing positive stress gradients.

To sum up, starting from M2, the stress direction alternates between compressive stress in metal layers and tensile stress in via layers. This effect opens a possibility to compensate many stress-related issues on design level. By proper structuring the metal and via layers in multi-layer structures, different device curvatures could be obtained by design.

#### 4.4.2. Single- and Multi-Metal Plates Curvature Comparison

More detailed curvature examination has been done using Sensofar (Terrassa, ES) Plu Neox confocal profiler. We examined several *z*-axis devices made of different metal layers or their combination and estimated the radius of curvature *R*, using Equation (2):

(2) $$R\approx \frac{L^{2}}{8\Delta z}$$

where *L* is a arc length and Δz is a difference between a position of the arc vertex and ideally flat plate.

**Figure 11:**
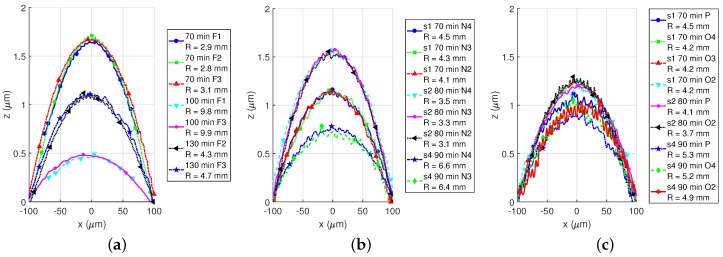
Plate curvature comparison: TM1 plates on *Accused* (**a**) , TM2 plates (**b**) and TM2-TVia2-TM1 plates from the same samples on *Bailed* (**c**). Data were clipped on 100 μm radius from a plate center.

In Figure 11a, a comparison of several TM1 plates are depicted. Three *Accused* chips of different etching time were examined, and depending on availability, two or three different devices of different plate diameter were measured at each chip. As can be seen, there is a significant dispersion of *R* between different chips: 2.9 mm–3.1 mm for 70 min sample and 9.7 mm–9.8 mm for 100 min sample. On the other hand, the curvature exhibits very small on-chip variation that, furthermore, may come from measurement error or the inaccuracy of Equation (2).

TM2 plates exhibited a similar behaviour (see Figure 11b), although showed less chip-to-chip variation. The compared devices came from *Bailed* chip. For the same N3 devices, the radius of curvature varied from 3.3 to 6.4 mm, while the variation between different devices on the same chip remained small in each case.

The best results in terms of chip-to-chip variations were exhibited by thick TM2-TVia2-TM1 plates (see Figure 11c). For a set of seven samples, the curvature of TM2-TVia2-TM1 plates follows the curvature of standalone TM2 plates (Figure 10b). In none of the cases above, any meaningful dependency between etching time and device curvature has been noticed.

Furthermore, in Figure 10a, it can be seen how adding a via mesh on top of M3 can shape its curvature. M3 plate is bent up with R=1.7 mm, while three M3-TVia1 plates have the opposite bending with −2.9 mm∼−3.5 mm range. By modifying the via mesh density different curvature could be obtained.

#### 4.4.3. Design-Space Limitations Due to Residual Stress

Using the worst case radius of curvature obtained for each of the stacks studied in Section 4.4.2, we can estimate the maximum useful dimensions of a device for a given layer configuration. Considering a device made of stack with thickness *h* and radius of curvature *R* and suspended over a bottom fixed plate with a vertical gap *d*, we can define the pass/fail criteria for the studied structures. Assuming that a device is suspended on its perimeter, the criteria are more strict for convex (R<0) stacks as too large device would touch the bottom surface or could be susceptible to pull-in. In the case of concave (R>0) shapes, the main issues would be the finger misalignment for the lateral devices, and an excessive gap raise in the center for the vertical devices.

The criteria in Table 6 are applied to selected layer configurations in order to calculate the maximum device diameter:

(3) $$L_{\max}\approx \sqrt{8R\Delta z_{\max}}$$

In the case of the square-shaped *z*-axis device, $L_{\max}$ corresponds to the diagonal of the plate. While square plates are convenient from the area utilization point of view, if the maximum performance is targeted (i.e., maximum mass and sensing area), a circular shape is preferred. This concept is exploited in case of Device P, which has a diameter of 360 μm close to the maximum allowed for TM1-TM2 stack suspended on top of M1 bottom plate. Similar device size and the same layer configuration was also chosen for the final lateral device. The results TM1-TM2 as well as the other tried stacks are presented in Table 7.

### 4.5. Anti-Stiction Measures

Stiction is one of the main failure mechanism in wet-etched surface micromachined devices. On the processing level, typically CO2 critical-point drying would be used to minimize its impact. In the case of our in-house post process, we have only used the rinse with IPA and methanol, which have very low surface tension, that reduce the sticking forces, though does not eliminate them entirely. Therefore, additional design level measures were taken to improve stiction robustness. First of all, moderate gaps were used and in case of the out-of-plane devices skipping at least of one metal layer was found to be necessary. In the case of lateral devices, distances between the fingers were chosen to be rather bigger than those forced by standard DRC rules.

#### Dimples

**Figure 12:**
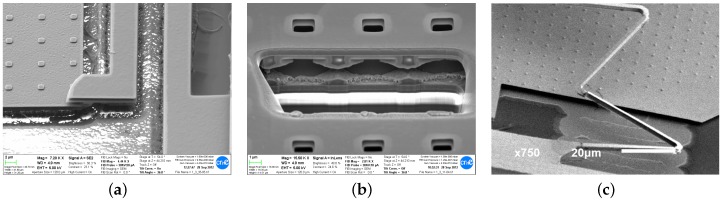
Different implementations of dimples using: MIM (**a**); oxide residues (**b**); and vias (**c**).

In a conventional MEMS surface-micromachined process such as PolyMUMPs, dimples are used to reduce the contact surface, thereby reducing the stiction issues. We have tried three methods to generate similar structures:

- *MIM dimples* were implemented as an array of minimum size MIM capacitors. They can be used only above M2. Due to their small height, they did not work properly in all cases and some devices remained stacked despite having MIM dimples. They are shown in Figure 12a.
- *Oxide dimples* appear when a specific etching time is used, so that a device is already released, while the oxide is not yet removed entirely from the space between a suspended and fixed plate, leaving dimples in the area most distant from the etching holes. Oxide dimples were found to be working correctly, however, due to high sensitivity to etching time and perforation holes density, they are not a very convenient solution. On the other hand, they may be useful as a short-circuit protection. They are shown in Figure 12b.
- *Via dimples*—implemented as a low-density, via array without metal on top—were found to be the most robust and reliable solution. Their performance was found to be independent on the etching time and even for the longest times they remained firmly attached to the bottom surface and practically no devices that used via dimples were found stuck to the bottom plate. The only potential inconvenience is that skipping at least one metal layer between the rotor and bottom surface is mandatory. They are shown in Figure 12c.

The capillary forces apparent during the wet etching and drying process that cause stiction problems still likely affects the devices even if dimples are used. In Figure 15c, profiles of circular devices (P) from seven different *Bailed* chip samples are depicted. While it can be seen that the devices have relatively stable plate shape, there is a significant gap dispersion between the samples. No correlation between the gap and etching time has been observed.

## 5. Selected Examples of Successfully Released Devices

In this section, we present a rapid overview of some most notable successfully released devices. The Comsol modal simulations were performed using a homogeneous solid model with E=65 GPa and the density adjusted to take into account the holes and the tungsten via impact on the overall device mass. Impedance measurements were performed in room temperature and vacuum using Agilent (Palo Alto, CA, USA) 4294A impedance analyzer and MMR Technologies (San Jose, CA, USA) LTMP-4 microprobe setup. For all devices, we can see C-V measurement, as well as magnitude and phase of total impedance. The resonance frequency is taken from the zero phase crossing of a virtual element that consists of the measured DUT with subtracted parallel capacitance $C_{0}$. For the sake of clarity, the magnitude of impedance is normalized to the value at the end of the frequency scale.

### 5.1. Z-Axis Devices

**Figure 13:**
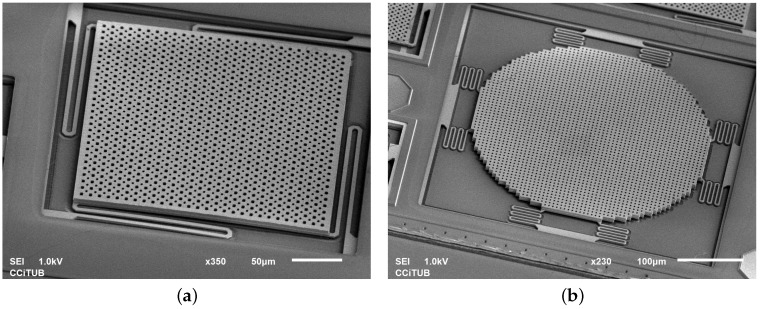
Selected *Z*-axis devices on on *Bailed* chip: 04 (**a**); and P (**b**).

**Figure 14:**
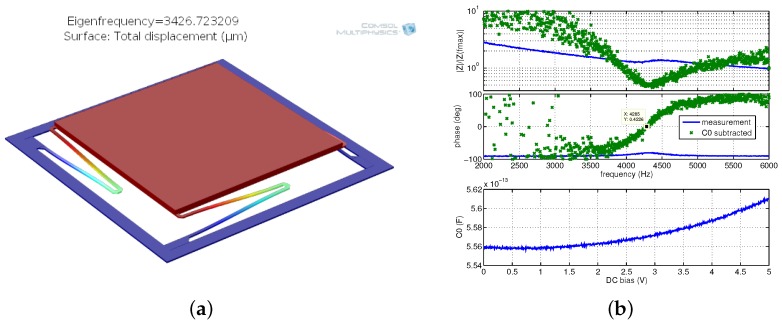
Device 04: modal simulation indicating eigenfrequency at 3.43 kHz (**a**); and impedance measurements with resonance at 4.29 kHz (**b**).

In Figure 13a, an SEM image of device O4 is presented. It showed the lowest measured resonance frequency of 4285 Hz (Figure 14b). The modal simulation (Figure 14a) indicates 3427 Hz eigenfrequency. Slight discrepancy is caused by the inaccuracy of the model that does not take into account effects such as hard Ti/TiN layers.

**Figure 15:**
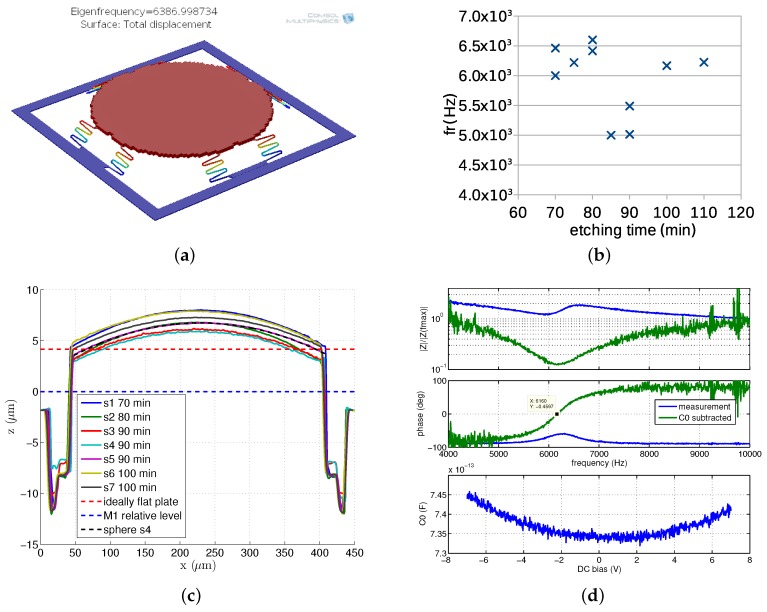
Circular *z*-axis accelerometer (P) on *Bailed* chip: modal simulation indicating eigenfrequency at 6387 Hz (**a**); measured resonance frequencies of several samples obtained after different etching time (**b**); profiles of several samples (**c**); and example of impedance measurement indicating resonance at 6160 Hz (**d**).

In Figure 13a, SEM image of circular *z*-axis accelerometer (P) on *Bailed* chip is presented. Simulations and measurements can be found in Figure 15. This device is the biggest one (360 μm diameter and 4.3 μg mass) that has been successfully released and its measurements over several samples revealed very stable results. Its slightly improved version has been integrated with electronics on the final chip.

### 5.2. Lateral Devices

**Figure 16:**
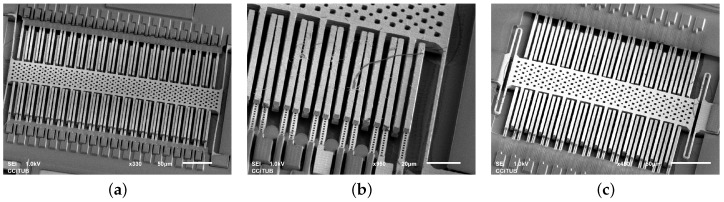
Lateral devices on *Bailed* chip: Buckling of TM2 fixed-fixed suspension in device T3 (**a**)—yet more evidence of compressive stress in TM2 layer. Short-circuit due to lateral stiction and peeling of Ti/TiN (**b**) were the main failure mechanisms of stator comb. The passivation over TVia2 and TM2 was damaged as well. Device T1 (**c**) was the first successfully measured (see Figure 17) lateral device.

**Figure 17:**
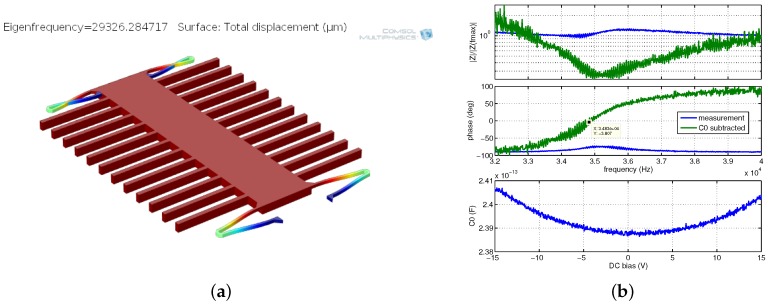
Device T1: modal simulation indicating eigenfrequency at 29.3 kHz (**a**); and impedance measurements with resonance at 34.8 kHz (**b**).

The lateral devices required more iterations to obtain functional devices. In Figure 16a,b SEM images of different failed prototypes are presented, showing issues such as buckling, lateral stiction, Ti/TiN peeling and passivation damage. In Figure 16c and Figure 17, the first successfully measured device is presented exhibiting resonance frequency at 34.8 kHz. Such a high value disqualifies it as a functional low-G acceleration sensor. Furthermore, several structural issues due to anchoring asymmetry as well as other reliability problems such as Ti/TiN peeling were observed.

**Figure 18:**
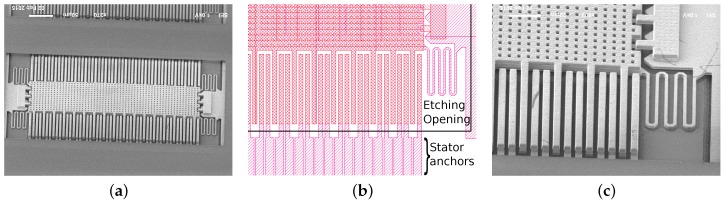
Final lateral device (adapted from [8] with permission, *©* 2015 IEEE): SEM image (**a**); in the layout, symmetric anchors of stator fingers can be seen (**b**). Closeup view (**c**) shows no mismatch between the stator fingers as well as no passivation damage and no Ti/TiN peeling that were the main issues in the initial designs in Figure 16.

All those problems have been addressed in the final device (see Figure 18) that used symmetric anchoring, no TM2 outside the passivation opening, had Ti/TiN removed and sizing allowing to reach resonance frequency of 7.4 kHz and noise floor below 200 μG/$\sqrt{Hz}$ limited by the sensing electronics [8].

### 5.3. Performance Comparison

**Figure 19:**
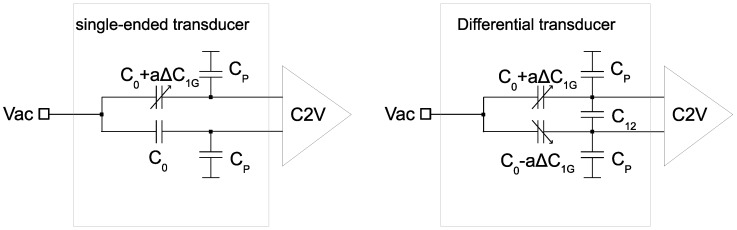
Generic circuits illustrating *FOM* calculation of single-ended (*z*-axis) and differential (*x*/*y*) sensors.

To quantify and compare the performance of different sensor prototypes, two figures of merit (*FOM*) are calculated. In Figure 19, generic circuits used for the *FOM* definitions for single-ended (*z*-axis) and differential (*x*/*y* axis) capacitive sensors are depicted. $C_{0}$ stands for the sensor capacitance with no acceleration, $\Delta C_{1G}\approx C_{0}/\left(\omega_{0}^{2}d\right)$ is the capacitive absolute sensitivity (assuming parallel plate approximation), $C_{p}$ is parasitic capacitance seen at the sensing nodes and $C_{12}$ is a parasitic capacitance between two sensing nodes, which appears only in the case of lateral sensors as it comes from the stator fingers overlap.

The first *FOM* is defined as a relative transducer sensitivity per one *G* that is represented by a ratio of the device sensitivity to the total capacitance seen at the sensing node:

(4) $$FOM=\frac{n\Delta C_{1G}}{C_{0}+C_{p}+2C_{12}}$$

where n=1 for single ended and n=2 for differential sensors. In any type of sensing front-end circuit, the parallel capacitance impacts the performance by either attenuating the signal or amplifying the noise [23]. Therefore, such a definition allows comparing SNR between different transducers.

*FOM2* is defined as relative sensitivity taking into account the sensing capacitance only:

(5) $$FOM2=\frac{n\Delta C_{1G}}{C_{0}}=\frac{n}{\omega_{0}^{2}d}$$

In other words, it is an intrinsic sensitivity of a device and a theoretical limit of *FOM* if the absence of parasitics. Having both *FOM* and *FOM2* shows the impact of the parasitics on the sensor. In general, *FOM2* should be as big as possible, however there is an important sensitivity-linearity trade-off, especially for single-ended sensors. Other factors constraining its maximum value are the sensor dynamic range and pull-in voltage.

#### FOM Calculation

In Table 8, the summary of *FOM* calculation is presented. The parasitic capacitance values $C_{p}$ and $C_{12}$ were obtained from Assura C-only extraction. For O2–O4 and *P* devices, *d*, $C_{O}$, $f_{0}$ and $\Delta C_{1G}$ were estimated from the C-V and resonance measurements taking into account the capacitor curvature and the electrostatic spring softening effect.

The results show a clear area-sensitivity trade-off. An improvement could be achieved in two ways. The first strategy would consist in increasing *FOM2*, by further pushing down the resonance frequency and decreasing the gap. This however can be risky from device yield and reliability point of view. In addition, the Brownian noise can become a limiting factor in this scenario. The other strategy could consist in improving *FOM* by optimizing the parasitic capacitance. It can be achieved by replacing the common support frame with individual anchors optimized for minimum area required to maintain the device on its place. In the case of the lateral sensor, the sizing of the stator anchors could be trimmed as well.

## 6. Conclusions

In this paper, we have presented MEMS integration experiments in IHP 0.25 μm CMOS technology. On the example of three subsequent chips, we have shown a prototyping process from design space exploration through design refinement until the development of functional CMOS-MEMS accelerometer with resonance frequencies down to 4.3 kHz, mass up to 4.3 μm and rotor diameters up to 360 μm. Although the academic scale of the experiment does not allow concluding meaningful yield of the post-processing, in a qualitative way, we have demonstrated how subsequent design generations solve the main manufacturing issues. We strongly believe that this article will allow the other researchers and designers to tighten the learning curve necessary to successfully develop CMOS-MEMS devices in similar technologies and that the presented design tricks and techniques will help them to avoid several structural and reliability problems such as layer delamination, stiction, passivation fracture and device cracking due to stress, to name the most important ones.

## Figures and Tables

**Table 1:** Layer composition of *z*-axis devices on *Accused*, *Bailed* and *Released* chips. The typical failures are: device Stiction, Delamination, Fracture, Curling and Peeling of Ti/TiN or passivation.

Chip	Group	Top	Bottom	Susp.	Typical Failures				
					*S*	*D*	*F*	*C*	*P*
*A*	A, G	M3	M2	M3	×		×	×	
	B, C	M3	M1	M3			×	×	
	D	M3-TM1	M2	M3	×	×	×	×	
	E	TM1	M3	TM1	×				
	F	TM1	M2	TM1				
	H	M3-TM1	M1	M3		×	×	×	
	I1	TM2	TM1	TM1					×
	I2	M3-TM1-TM2	M1	M3		×	×	×	×
	I3	M3-TM1-TM2	M2	M3	×	×	×	×	×
*B*	N1	TM2	TM1	TM2					×
	N2	TM2	M3	TM2					×
	N3	TM2	M2	TM2					×
	N4	TM2	M1	TM2					×
	O2	TM1-TM2	M2	TM1					×
	O3	TM1-TM2	M2	TM1					×
	O4	TM1-TM2	M1	TM1					×
	P	TM1-TM2	M1	TM1					×
	*R*	Z	TM1-TM2	M1	TM1					
R		TM1-TM2	M2	TM1					

**Table 2:** Layer composition of lateral devices on *Accused*, *Bailed* and *Released* chips. The typical failures are: device Delamination, Curling, Buckling and Peeling of Ti/TiN or passivation.

Chip	Device	Rot. Plate	Susp.	Rot. Finger	Stat. Finger	Typical Failures			
						*D*	*C*	*B*	*P*
*A*	L1, 2, 10	M3	M3	M3	M2-M3-TM1	×	×		
	L3, 8	M3-TM1	M3	M3	M2-M3-TM1	×	×		
	L4	M3-TM1-TM2	M3	M3	M2-M3-TM1	×	×		×
	L5	M3-TM1-TM2	M3	M3-TM1	M2-M3-TM1	×	×		×
	L6, 9	M2-M3-TM1	M3	M2-M3	M2-M3-TM1	×			
	L7	M2-M3	M3	M2-M3	M2-M3-TM1	×			
*B*	T1	TM1-TM2	TM2	TM1-TM2	TM1-TM2				×
	T2	TM1-TM2	TM2	TM2	TM1-TM2				×
	T3	TM1-TM2	TM2	TM1-TM2	TM1-TM2			×	×
*R*	X	TM1-TM2	TM1	TM1-TM2	TM1-TM2				

**Table 3:** Approximate release depth vs. time.

t [min]	TM2	TM1	M3	M2	M1	Si	Depth
40	R	C	C	C	C	C	∼4 μm
60	R	E	C	C	C	C	∼6 μm
80	R	R	E	E	C	C	∼8 μm
100	R	R	R	E	C	C	∼10 μm
120	R	R	R	R	R	E	∼12 μm
140	R	R	R	R	R	R	∼14 μm
Legend	Released		Exposed		Covered		

**Table 4:** Comparison of observed metal-via-metal stack performance for different via patterns and metal layers: integrity of the mechanical connection and preservation of the silicon oxide enclosed by vias.

	Array	Bars	Mesh	Array	Bars	Mesh
	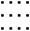	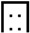	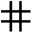	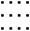	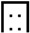	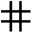
	Integrity			Oxide		
M1-M2	*√*	*√*	*√*	n/a	*√*	*√*
M2-M3	*√*	*√*	*√*	n/a	*√*	*√*
M3-TM1	×	×	∼	n/a	×	×
TM1-TM2	×	×	*√*	n/a	×	×

**Table 5:** Qualitative summary of observed stress ($\sigma_{0}$) and stress gradient ($\sigma_{1}$) for subsequent metal and via layers.

	M1	Via1	M2	Via2	M3	TVia1	TM1	TVia2	TM2
$\sigma_{0}$	>0	>0	<0	>0	<0	>0	<0	>0	<0
$\sigma_{1}$	>0		<0		<0		<0		<0

**Table 6:** Pass/fail criteria to estimate the maximum MEMS dimensions for a given curvature type and geometry.

Device	Curvature	$|\Delta z_{\max}|$	Failure Type
lateral	⌢(R>0)	h/2	misalignment
lateral	⌣(R<0)	min(h/2,d)	misalignment or collapse
z-axis	⌢(R>0)	*d*	excessive gap raise
z-axis	⌣(R<0)	d/2	collapse, excessive gap decrease

**Table 7:** Estimated maximum device size $L_{\max}$ for selected layer configurations. The values without specified unit are in microns.

Stack	$R_{\mathrm{wc}}$ (mm)	Bottom	*h*	*d*	Lateral		*Z*-axis	
					$|\Delta z_{\max}|$	$L_{\max}$	$|\Delta z_{\max}|$	$L_{\max}$
M3	1.7	M2	0.73	0.9	0.37	70	0.9	111
	1.7	M1	0.73	2.53	0.37	70	2.53	185
M3TV1	−2.9	M1	1.63	0.9	0.82	138	0.45	102
	−2.9	M2	1.63	2.53	0.82	138	1.265	171
TM1	2.8	M3	2	0.9	1.00	150	0.9	142
	2.8	M2	2	2.53	1.00	150	2.53	238
	2.8	M1	2	4.16	1.00	150	4.16	305
TM1-TM2	3.7	M3	8	0.9	4.00	344	0.9	163
	3.7	M2	8	2.53	4.00	344	2.53	274
	3.7	M1	8	4.16	4.00	344	4.16	358
TM2	3.1	TM1	3	0.9	1.50	193	0.9	149
	3.1	M3	3	2.53	1.50	193	2.53	250
	3.1	M2	3	4.16	1.50	193	4.16	321
	3.1	M1	3	9.16	1.50	193	9.16	477

**Table 8:** Comparison of the *FOM* and area data of different sensors.

Device	$f_{0}$	*m*	$C_{0}$	*d*	$\Delta C_{1G}$	$C_{p}$	$C_{12}$	*FOM*	*FOM2*	Area
	kHz	μg	*fF*	μm	*fF*/*G*	*fF*	*fF*	$10^{-3}$	$10^{-3}$	mm${}^{2}$
02 [7]	20.6	0.6	65.6	2.94	0.014	245	0	0.045	0.21	0.055
03	13.48	1.25	118	3.75	0.056	288	0	0.14	0.47	0.076
04	4.35	2	130	5.6	0.41	367	0	0.83	3.15	0.123
*P*	6.63	4.3	200	6.75	0.26	650	0	0.31	1.31	0.25
*Y* [8]	7.4	1.95	84	3	0.13	270	450	0.2	3	0.18

## References

[1] Fedder G.K., Santhanam S., Reed M.L., Eagle S.C., Guillou D.F., Lu M.S.C., Carley L.R. (1996). Laminated High-aspect-ratio Microstructures in a Conventional CMOS Process. Sens. Actuators A Phys..

[2] Xie H., Erdmann L., Zhu X., Gabriel K.J., Fedder G.K. (2002). Post-CMOS Processing for High-Aspect-Ratio Integrated Silicon Microstructures. J. Microelectromech. Syst..

[3] Kaynak M., Valenta V., Schumacher H., Tillack B. MEMS module integration into SiGe BiCMOS technology for embedded system applications. Proceedings of the 2012 IEEE Bipolar/BiCMOS Circuits and Technology Meeting (BCTM).

[4] Verd J., Uranga A., Teva J., Lopez J., Torres F., Esteve J., Abadal G., Perez-Murano F., Barniol N. (2006). Integrated CMOS-MEMS with on-chip readout electronics for high-frequency applications. IEEE Electron Device Lett..

[5] Dai C.L. (2006). A maskless wet etching silicon dioxide post-CMOS process and its application. Microelectron. Eng..

[6] Fernández D., Ricart J., Madrenas J. (2010). Experiments on the Release of CMOS-micromachined Metal Layers. J. Sens..

[7] Michalik P., Sánchez-Chiva J., Fernández D., Madrenas J. (2015). CMOS BEOL-embedded z-axis accelerometer. Electron. Lett..

[8] Michalik P., Sánchez-Chiva J., Fernández D., Madrenas J. CMOS BEOL-Embedded Lateral Accelerometer. Proceedings of the 2015 IEEE Sensors.

[9] IHP (2014). SG25H1 Process Specifications.

[10] Xiao H. (2012). Chapter 11 Metallization. Introduction to Semiconductor Manufacturing Technology.

[11] Kaynak M. (2014). RF-MEMS Switch Module in a 0.25 μm SiGe: C BiCMOS Process. Ph.D. Thesis.

[12] Szaniawski K., Napieralski A., Sekalski P., Podsiadly P. Design of a prototype of a 3-axis capacitive acceleration sensor. Proceedings of the 24th International Conference on Microelectronics (IEEE Cat. No. 04TH8716).

[13] Fedder G.K., Chae J., Najafi K., Denison T., Kuang J., Lewis S., Kulah H. (2004). Advanced Micro and Nanosystems. Vol. 2 CMOS—MEMS.

[14] Michalik P., Fernández D., Madrenas J., Kaynak M., Wietstruck M. An Approach to MEMS Smart Sensor Design using CMOS BEOL. Proceedings of the 14th International Symposium on RF-MEMS and RF-Microsystems, MEMSWAVE.

[15] Banerji S., Michalik P., Fernández D., Madrenas J., Mola A., Montanyà J. (2017). CMOS-MEMS resonant pressure sensors: Optimization and validation through comparative analysis. Microsyst. Technol..

[16] Banerji S., Fernández D., Madrenas J. (2017). Characterization of CMOS-MEMS Resonant Pressure Sensors. IEEE Sens. J..

[17] Williams K., Gupta K., Wasilik M. (2003). Etch rates for micromachining processing-Part II. J. Microelectromech. Syst..

[18] Valle J., Fernández D., Madrenas J. (2016). Experimental Analysis of Vapor HF Etch Rate and Its Wafer Level Uniformity on a CMOS-MEMS Process. J. Microelectromech. Syst..

[19] Michalik P., Fernández D., Madrenas J. (2016). Integrated Circuit Comprising Multi-Layer Micromechanical Structures with Improved Mass and Reliability by Using Modified Vias and a Method to Obtain Thereof. U.S. Patent.

[20] Fang W., Wickert J. (1996). Determining mean and gradient residual stresses in thin films using micromachined cantilevers. J. Micromech. Microeng..

[21] Paul O., Ruther P. (2005). Advanced Micro and Nanosystems. Vol. 2 CMOS—MEMS.

[22] Valle J., Fernández D., Madrenas J., Barrachina L. (2017). Curvature of BEOL Cantilevers in CMOS-MEMS Processes. J. Microelectromech. Syst..

[23] Yazdi N., Kulah H., Najafi K. Precision readout circuits for capacitive microaccelerometers. Proceedings of the 3rd IEEE Conference on Sensors.

